# ClotPro viscoelastic testing in coagulation disorders in dogs: a retrospective analysis

**DOI:** 10.1093/jvimsj/aalag254

**Published:** 2026-10-09

**Authors:** Pavlos G Doulidis, Carolina Frizzo-Ramos, Larissa J Prankch, Barbara Rütgen, Iwan A Burgener, Nicole Luckschander-Zeller

**Affiliations:** Clinical Unit of Small Animal Internal Medicine, Department for Companion Animals and Horses, University of Veterinary Medicine, 1210 Vienna, Austria; Clinical Unit of Anesthesiology and Perioperative Intensive Care, Department for Companion Animals and Horses, University of Veterinary Medicine, 1210 Vienna, Austria; Clinical Unit of Small Animal Internal Medicine, Department for Companion Animals and Horses, University of Veterinary Medicine, 1210 Vienna, Austria; Department of Pathobiology, University of Veterinary Medicine, 1210 Vienna, Austria; Clinical Unit of Small Animal Internal Medicine, Department for Companion Animals and Horses, University of Veterinary Medicine, 1210 Vienna, Austria; Clinical Unit of Small Animal Internal Medicine, Department for Companion Animals and Horses, University of Veterinary Medicine, 1210 Vienna, Austria

**Keywords:** fibrinolysis, hemostasis, hypercoagulability, thromboelastography

## Abstract

**Background:**

Viscoelastic testing provides whole-blood assessment of coagulation, but clinical data on the performance of the ClotPro in dogs with naturally occurring diseases are limited.

**Hypothesis/Objectives:**

To evaluate pathway coherence of ClotPro variables with routine coagulation tests and to explore disease-associated viscoelastic phenotypes, fibrinolysis, and hypercoagulability.

**Animals:**

Eighty-four client-owned dogs admitted to a university hospital.

**Methods:**

Retrospective cohort study. ClotPro variables (clotting time, amplitude at 10 min, maximum clot firmness, maximum lysis across extrinsic pathway test [EX], intrinsic pathway test [IN], fibrinogen test channel, aprotinin test [AP] channels) were compared with prothrombin time (PT), activated partial thromboplastin time (aPTT), platelet count (PLT), and D-dimer using Spearman correlation with Benjamini–Hochberg adjustment. Group differences were assessed with Kruskal–Wallis and Dunn’s post hoc. Hyperfibrinolysis (HF) was defined using paired EX–AP. A hypercoagulability index (HCI) and platelet/fibrin-adjusted residual firmness were derived.

**Results:**

PT correlated with clotting time in EX channel (ρ = 0.48, *q* = 0.002) and aPTT with clotting time in IN channel (ρ = 0.42, *q* = 0.007). PLT correlated with maximum clot firmness in EX channel (EX_MCF) (ρ = 0.50, *q* = 0.001). maximum clot firmness in FIB channel correlated with EX_MCF (ρ = 0.58, *q* < 0.001) and maximum clot firmness in IN channel (ρ = 0.47, *q* = 0.003). maximum lysis in EX channel (EX_ML) correlated inversely with EX_MCF (ρ = −0.35, *q* = 0.028). D-dimer correlated with EX_ML (ρ = 0.30, *q* = 0.049). Group-level distributions overlapped substantially. HF was infrequent among cases (33% in dogs with hepatopathy and 21% in dogs with hemorrhagic diarrhea). The HCI differed across diseases (*P* = .0002), whereas platelet/fibrin-adjusted residual firmness did not.

**Conclusions and clinical importance:**

ClotPro variables demonstrated pathway-specific coherence with routine coagulation testing and consistent fibrinolysis patterns. Disease states did not define discrete viscoelastic phenotypes, supporting case-level interpretation.

## Introduction

Coagulation disorders are common in veterinary medicine and if not promptly diagnosed and treated can become life-threatening. The routine secondary hemostatic tests usually performed in clinical practice are prothrombin time (PT), activated partial thromboplastin time (aPTT) and thrombin time (TT). These tests are useful for diagnosing many conditions, but they do not provide holistic information about clot formation, as they do not evaluate specific molecular and cellular surface interactions. Furthermore, these variables are not reliable in diagnosing prothrombotic or hypercoagulable states. Many diseases are associated with an increased thrombotic risk, which significantly increases morbidity and case fatality rate.^1^ Viscoelastic testing serves as a valuable tool for assessing the whole blood hemostasis, thereby informing therapeutic decisions and guiding the initiation of thromboprophylactic or thrombolytic treatment.^2^^,^^3^ Viscoelastic testing offers a wide evaluation of all stages of the coagulation cascade, from initiation to amplification and propagation up to fibrinolysis and dissolution of the clot.^4^^,^^5^ ClotPro (Haemonetics Corporation) is a modern viscoelastic whole-blood coagulation analyzer that, unlike other viscoelastic devices, offers standardized pipette tips prefilled with distinct reagents, enabling a wide range of specific assays.^6^ ClotPro analysis utilizes citrated whole blood 30 min after collection, and employs active tip technology during pipetting, with reagents preloaded in pipette tips.^7^ This approach ensures accurate sample reconstitution and reduces the risk of preanalytical error. After activation, the blood adheres to both the cup and pin surfaces, and clot strength is measured continuously and displayed graphically. The analyzer includes several reagents (active tips) that enable detection and assessment of primary and secondary hemostasis, factor deficiencies, fibrinogen, fibrinolysis, heparin, and direct oral anticoagulant effects, and antifibrinolytic drug response.^8^ Recombinant tissue factor is used to evaluate the coagulation process with extrinsic activation (EX Test), ellagic acid for intrinsic pathway (IN Test), while functional qualitative detection of the fibrinogen level (FIB Test) is achieved by dual inhibition of the platelets by cytochalasin D and a synthetic fibrinogen receptor agonist. The device features 6 channels, allowing multiple tests to be conducted simultaneously. In contrast to other systems such as rotational thromboelastometry and thromboelastography, ClotPro provides robust fibrinolysis assessment using the direct plasmin inhibitor aprotinin (AP Test) and standardized, drug-specific assays.^9^ AP channel serves as a native “lysis-OFF” control. Comparing EX vs AP allows confirmation of true fibrinolysis and exclusion of preanalytical mistakes, without extra preparation. ClotPro is successfully used in human medicine for the assessment of bleeding risk or hypercoagulability and thrombotic risk in various disorders.^8^^,^^10–18^ Although data in canine medicine are limited, canine specific reference ranges for the ClotPro analyzer are published.^19^ Data on cats are even more scarce, but predict reliable performance.^20^ Aim of this retrospective study was to assess correlation of ClotPro variables with routine coagulation testing and to evaluate the performance of the ClotPro viscoelastic analyzer across various diseases, linked with either increased thrombosis risk or bleeding tendency.

## Materials and methods

This was a single center retrospective study between 2022 and 2024. Dogs were eligible if they had a valid ClotPro analysis including at least EX Test and IN Test assays, optimally also FIB Test and AP Test, as well as sufficient concurrent routine coagulation tests (PT, aPPT) and platelet count (PLT) obtained within 30 min after collection. Cases were assigned to a single primary disease category a priori based on clinician diagnosis and record review: acute hemorrhagic diarrhea syndrome (AHDS), bleeding (non-AHDS), hyperadrenocorticism (Cushing), hepatopathy, immune-mediated hemolytic anemia (IMHA), thrombosis of unclear etiology, protein-losing nephropathy (PLN), protein-losing enteropathy (PLE), or thrombocytopenia. Dogs with incomplete records, obvious sample mishandling, or duplicate measurements from the same visit were excluded. From ClotPro traces, we extracted: clotting time (CT, s), amplitudes at 5/10/20 min (A5/A10/A20, mm), maximum clot firmness (MCF, mm), and maximum lysis (ML, %) for EX, IN, FIB, and AP where available.

### Fibrinolysis assessment

The burden metrics were calculated as ΔML = EX_ML − AP_ML and AP_MCF − EX_MCF (mm), suggesting loss of firmness due to lysis. A case was flagged as hyperfibrinolysis (HF) if (EX_ML ≥ T1 and AP_ML ≤ T2) OR (ΔML ≥ T3), with default thresholds T1 = 15%, T2 = 5%, T3 = 10% unless otherwise specified. HF prevalence analyses were restricted to runs with both EX and AP available.

### Hypercoagulability index and platelet/fibrin-adjusted firmness

For the composite hypercoagulability index (HCI) we combined faster initiation (shorter CT) and greater firmness (A10/MCF) across EX/IN into a single standardized score so that higher values reflect a more pro-coagulant phenotype. For each metric 𝑥 we computed a robust z-score across the cohort. We additionally estimated the portion of EX_MCF not explained by platelets and fibrin contribution (FIB_MCF) expressed as the Platelet/fibrin-adjusted firmness.

### Statistical analysis

Comparisons between disease groups for selected metrics (EX_CT, IN_CT, EX_MCF, IN_MCF, fibrinolysis metrics EX_ML, AP_ML, ΔML) were performed with Kruskal–Wallis test with Dunn’s post-hoc; Benjamini–Hochberg false discovery rate (FDR) adjustment was applied within each family of hypotheses. The Spearman ρ was used for correlations followed with regression analysis. Hypercoagulability was evaluated in 3 ways: (1) strict rule (high MCF and/or low CT using cohort 90th/10th percentiles), (2) HCI (continuous and ≥ 90th percentile), and (3) platelet/fibrin-adjusted residual (continuous and ≥ 90th percentile). A 2-sided FDR-adjusted *P* < .05 (*q*-value) was considered statistically significant.

## Results

Data from a total of 84 dogs were used for retrospective analysis. The median weight of the cohort was 17.6 kg (range 1.60-60.00 kg) and the median age was 11.3 years old (range 1.8-15.3 years old). Of these dogs 38% (*n* = 32) were female spayed, 23% (*n* = 19) were female intact, 23% (*n* = 19) were male neutered and 16% (*n* = 14) were male intact. The frequency of the top 10 breeds in the cohort is demonstrated in Figure 1. Of these retrospective cohort, the most prevalent diseases were AHDS (*n* = 14, 17%), hepatopathy (*n* = 9, 11%), thrombocytopenia (*n* = 7, 8%), PLN (*n* = 5, 6%), thrombosis (*n* = 4, 5%), IMHA (*n* = 4, 5%), PLE (*n* = 3, 4%), neoplasia (*n* = 3, 4%), bleeding (*n* = 3, 4%), sepsis (*n* = 3, 4%), and other diseases with less than 2 cases each. The median PLT count was 187 × 10^3^/μL (Range 13-682 × 10^3^/μL) while the median hematocrit count was 40.5% (Range 14%-64%).

**Figure 1:**
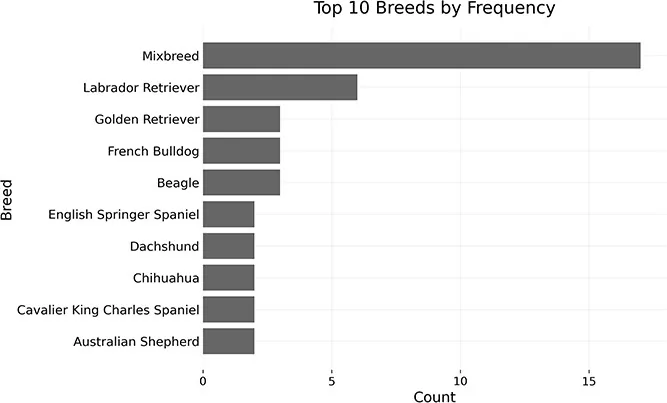
Histogram demonstrating the count of mixbreed dogs followed by the most common breeds by frequency in the cohort.

### Correlations of ClotPro variables with routine coagulation testing

To evaluate the agreement between ClotPro variables (CT, A10, MCF, ML across EX/IN/FIB/AP) and routine hemostatic testing (PT, aPTT, PLTs, D-dimer) we built a correlation matrix linking the above values (Figure 2). PT correlated positively with EX_CT (ρ = 0.48, *q* = 0.002), and aPTT with IN_CT (ρ = 0.42, *q* = 0.007), supporting path-specific convergence and confirming convergent validity. Platelet count correlated with viscoelastic firmness on the EX path; EX_A10 (ρ =0.55, *q* < 0.001) and EX_MCF (ρ =0.50, *q* = 0.001). FIB_MCF correlated positively with EX_MCF (ρ =0.58, *q* < 0.001) and IN_MCF (ρ =0.47, *q* = 0.003). Lysis showed the expected correlation with firmness as EX_ML correlated negatively with EX_MCF (ρ = −0.35, *q* = 0.028). D-dimer correlated positively with viscoelastic lysis (EX_ML: ρ = 0.30, *q* = 0.049; ΔML: ρ =0.33, *q* = 0.039). PLT had a moderate to strong correlation with both EX A10 (Spearman *r* = 0.55, *q* < 0.001) and EX MCF (*r* = 0.50, *q* = 0.001). The fibrin contribution, measured on the FIB channel, was also positively related to whole-blood firmness, showing correlations with EX MCF (*r* = 0.58, *q* < 0.001) and IN MCF (*r* = 0.47, *q* = 0.003).

**Figure 2:**
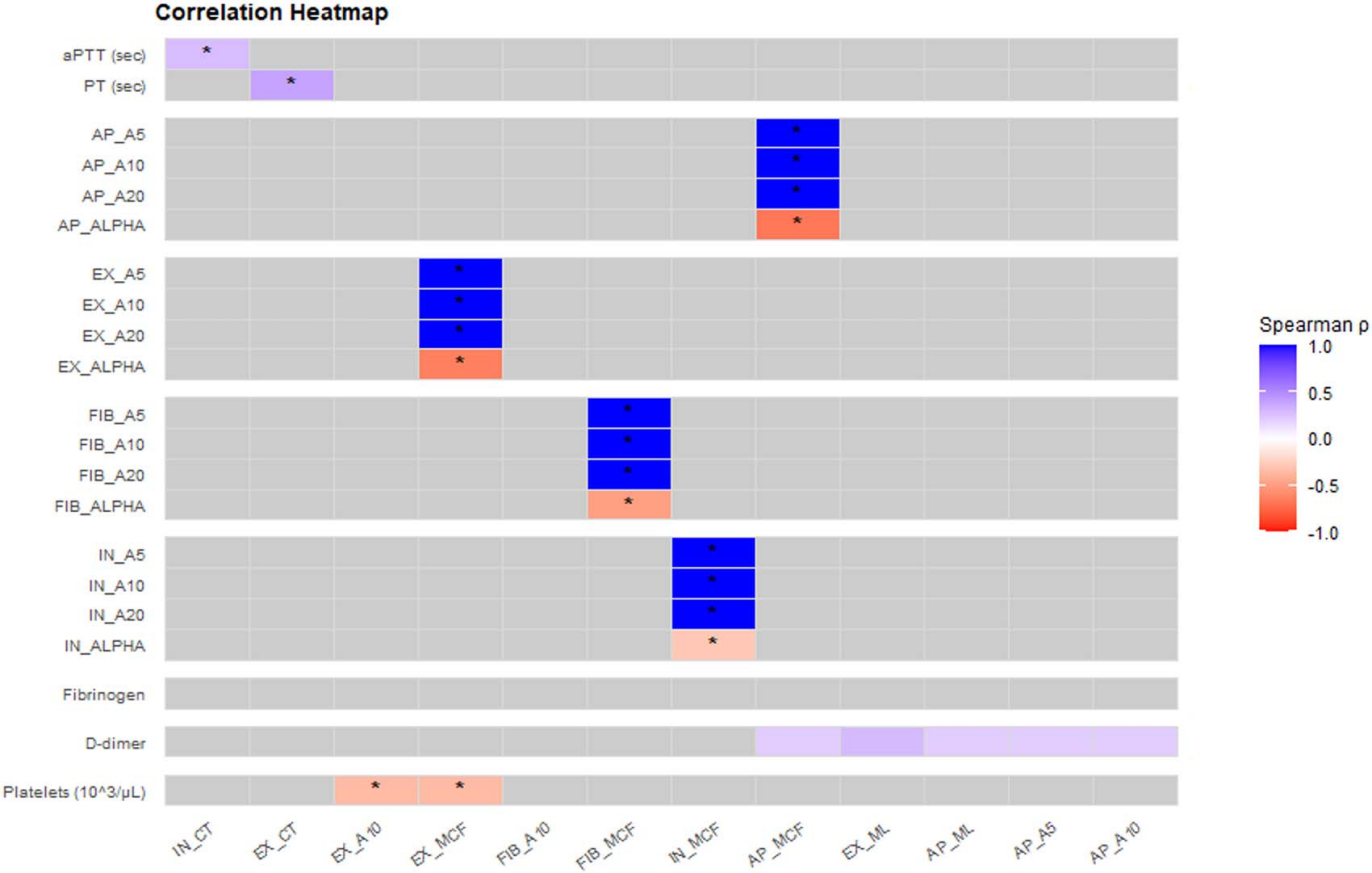
Correlation matrix (Spearman; BH/FDR across cells) linking routine plasma tests (PT, aPTT, PLTs, D-dimer) to ClotPro variables (CT, A10, MCF, ML across EX/IN/FIB/AP). Significant cells are starred in the heatmap; non-tested/missing pairs are gray.

### Coagulation initiation and clot strength across diseases

We then compared initiation (EX_CT, IN_CT) across prespecified diseases (Figure 3). Overall, group differences are detectable in CT and MCF but with overlapping distributions, indicating small–moderate effects rather than clean separation. Across diseases, EX_CT differed at the group level, but distributions overlapped substantially. Differences in the IN_CT were not significant between groups. The direction in the EX_CT was pathway-specific and biologically coherent, as groups expected to have factor delay showed prolonged CT (bleeding, thrombocytopenia) whereas pro-thrombotic phenotypes (IMHA, PLE, PLN, Cushing) showed normal-to-short CT. Significant pairwise contrasts were, indicating small effects rather than discrete separation. Comparison of clot strength (EX_MCF, IN_MCF) among groups yielded similar results with significant overlap (Figure 4). As anticipated, groups with platelet/fibrin deficits (acute bleeding, thrombocytopenia) tended toward lower MCF, while pro-coagulant states showed higher firmness (PLE, PLN, IMHA, Cushing). An important finding is that, consistent with assay mechanics, A10 tracked MCF closely, supporting reliable early interpretation. Because firmness is strongly influenced by platelets and fibrinogen, we also present a platelet/fibrin-adjusted residual in the hypercoagulability section to distinguish excess firmness from count/fibrin effects.

**Figure 3:**
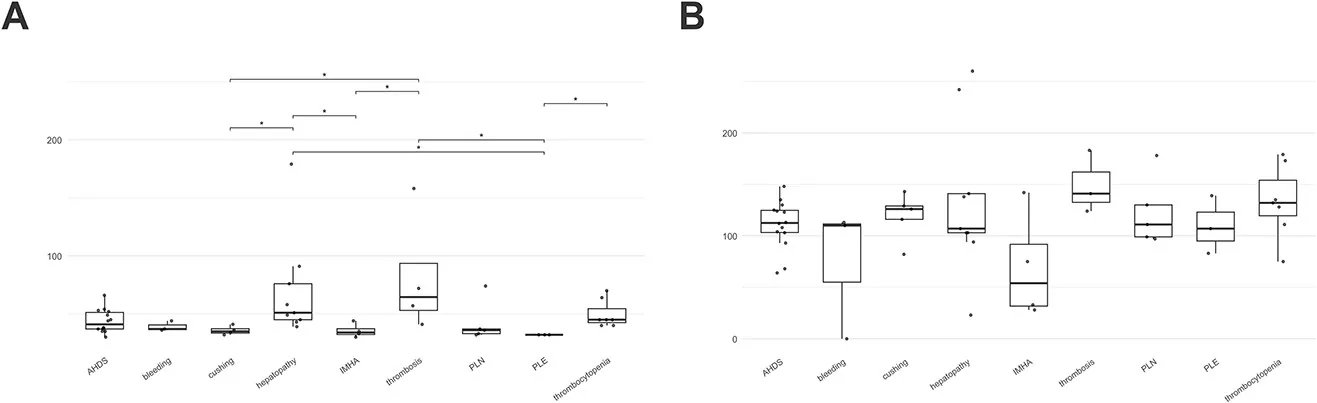
(A) Box-and-jitter plots demonstrating the comparison of the clot initiation time (CT sec) of the tissue factor activated pathway (EX_CT) between groups. (B) Box-and-jitter plots demonstrating the comparison of the CT (s) of the ellagic acid activated pathway (IN_CT) between groups. Asterisk (^*^) demonstrates pairwise significant statistical difference between groups (*q* < 0.05). Boxes show median and interquartile range (IQR); whiskers span 1.5 × IQR. Abbreviations: CT = clotting time; EX = extrinsic pathway; FDR = false discovery rate; IN = intrinsic pathway.

**Figure 4:**
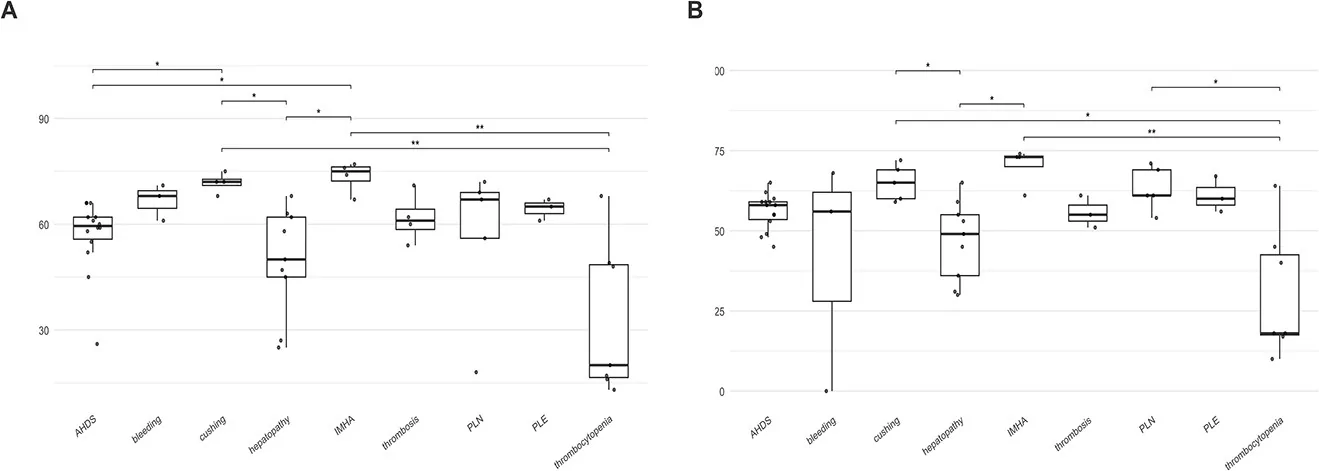
(A) Box-and-jitter plots demonstrating the comparison of the MCF (mm) of the tissue factor activated pathway (EX_MCF) between groups. (B) Box-and-jitter plots demonstrating the comparison of the MCF (mm) of the ellagic acid activated pathway (IN_MCF) between groups. Asterisk (^*^) demonstrates pairwise significant statistical difference between groups (*q* < 0.05). Boxes show median and interquartile range (IQR); whiskers span 1.5 × IQR. Abbreviations: MCF = maximum clot firmness; EX = extrinsic pathway; FDR = false discovery rate; IN = intrinsic pathway.

### Fibrinolysis assessment

From our cohort, EX_ML was missing in 15% of the cases (*n* = 8) and AP_ML in 48% of the cases (*n* = 26). Therefore, all fibrinolysis summaries and prevalence estimates were restricted to cases with both parameters available. Across diseases, AP_ML was generally low, supporting validity of EX-channel lysis and excluding preanalytical error (Figure 5). The median value AP_ML was AHDS 3.5% (95th Percentile 5.0%), bleeding 2.0% (95th Percentile 2.0%), Cushing 1.5% (95th Percentile 1.95%), hepatopathy 5.0% (95th Percentile 6.0%), thrombosis 4.0% (95th Percentile 4.9%), PLN 4.0% (95th Percentile 4.0%), PLE 3.0% (95th Percentile 4.8%), thrombocytopenia 2.5% (95th Percentile 3.85%). D-dimer correlated positively with viscoelastic lysis (EX_ML ρ = 0.30, *q* = 0.049; ΔM: ρ = 0.33, *q* = 0.039). As expected, higher lysis associated with lower final firmness on the same path (EX_ML vs EX_MCF ρ = −0.35, *q* = 0.028). These patterns are directionally coherent and support the biological validity of the EX–AP cointerpretation. HF was defined as EX_ML ≥ 15% with AP_ML ≤ 5%, or ΔML ≥ 10%. Using the eligible set (EX+AP present), HF events were infrequent across disease groups. Most notably HF was seen in 3 of 9 eligible cases of dogs with hepatopathy and 3 of 14 eligible cases of dogs with AHDS.

**Figure 5:**
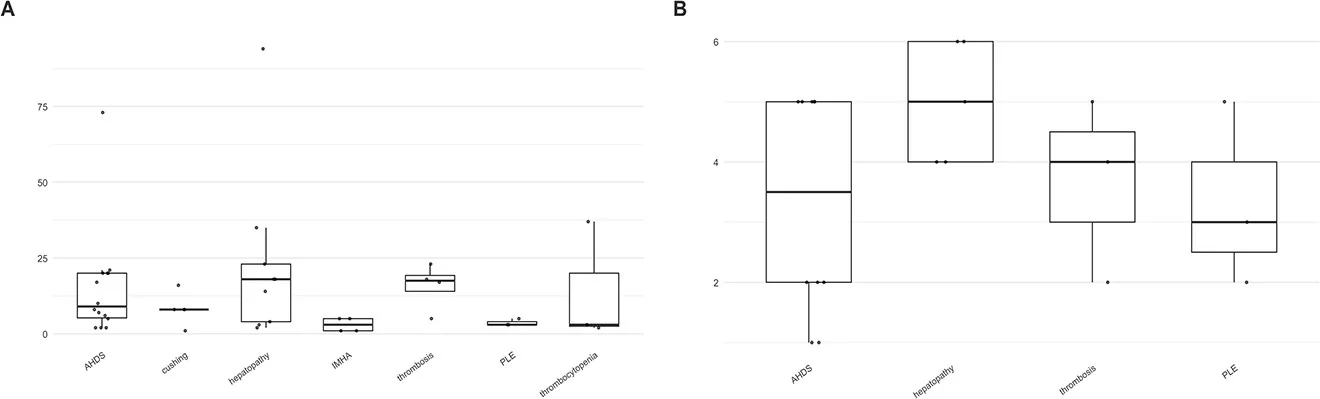
(A) Box-and-jitter plots demonstrating the comparison of the ML (%) of the tissue factor activated pathway (EX_ML) between groups. (B) Box-and-jitter plots demonstrating the comparison of the ML (%) of the AP_ML between groups. Asterisk (^*^) demonstrates pairwise significant statistical difference between groups (*q* < 0.05). Boxes show median and interquartile range (IQR); whiskers span 1.5 × IQR. Abbreviations: ML, maximum lysis; EX, extrinsic pathway; AP, aprotinin plasmin inhibition test; FDR, false discovery rate.

### Hypercoagulability index and platelet/fibrin-adjusted firmness

Hypercoagulability was summarized using a composite index that combined faster initiation (shorter CT) and greater firmness (A10/MCF) across EX and IN. Higher values indicate a more procoagulant phenotype. The index showed a right-tailed distribution (90th percentile 0.825). Across diseases, the index differed (*P* = 0.0002), with significant differences between some groups (Figure 6A). To separate clot architecture from engine effects, EX_MCF was adjusted for PLT and fibrin contribution (FIB_MCF), yielding a residual firmness measure. The residual showed a right-tailed distribution (90th percentile 10.679) and did not differed between diseases (*P* = .384) (Figure 6B).

**Figure 6:**
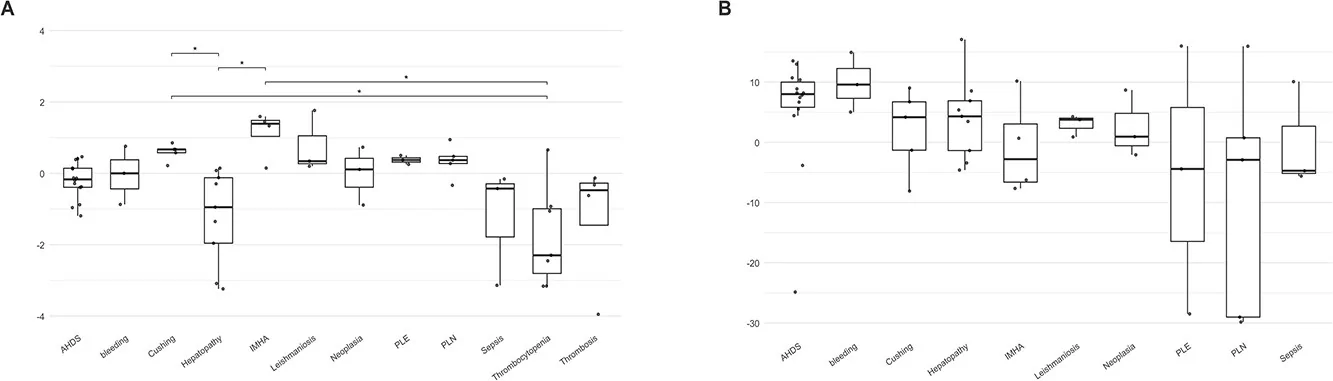
(A) Box-and-jitter plots of the composite hypercoagulability index (HCI), which combines faster initiation (shorter CT) and greater firmness (A10/MCF) across EX and IN pathways. (B) Box-and-jitter plots of the residual firmness derived from a cohort model of EX_MCF as a function of platelet count and fibrin contribution (FIB_MCF); values > 0 indicate excess firmness beyond platelets/fibrin. Boxes show median and interquartile range (IQR); whiskers span 1.5 × IQR. Asterisk (^*^) demonstrates pairwise significant statistical difference between groups (*q* < 0.05). Abbreviations: A10 = amplitude at 10 min; CT = clotting time; EX = extrinsic pathway; FDR = false discovery rate; IN = intrinsic pathway; MCF = maximum clot firmness.

## Discussion

In this retrospective study of a cohort of hospitalized dogs, ClotPro viscoelastic metrics demonstrated pathway coherence. PT correlates with EX_CT, while aPTT correlates with IN_CT. PLT and fibrin contribution were associated with clot firmness. Lysis measured on the EX-channel was inversely associated with firmness, and D-dimer exhibited a modest positive association with lysis. Group differences in CT and firmness were observed across diseases, although distributions overlapped and no reliable group separation can be assumed. HF was infrequent when assessed using paired EX and AP channels. A right-tailed hypercoagulable phenotype was identified using both a composite index and a PLT- and fibrin-adjusted firmness measure.

In agreement with a previous study on healthy dogs, EX_CT showed significant correlation with PT and IN_CT showed significant correlation with aPTT.^19^ Our results in this retrospective diseased cohort suggest that ClotPro offers excellent evaluation of the initiation phase of coagulation in both extrinsic and intrinsic pathways, considering the fact that it also considers cellular surface interactions.^21^ Studies in people report similar performance.^9^^,^^22^^,^^23^ An important finding of this study is the excellent predictive value and close tracking of the A5 amplitude with the MCF. In emergencies that require rapid medical or surgical intervention, this fact might be crucial, as it allows faster decision making, without waiting for the final value of the MCF. We also found that higher PLT numbers were linked to greater viscoelastic firmness. The strong correlation of PLT count with both EX_A10 and EX_MCF and the positive correlation of the FIB channel with the whole blood firmness show that clot strength in whole blood depends on both platelet number and fibrin(ogen) contribution. This supports using the EX and FIB comparison to assess the platelet component of firmness and also aligns with findings of previous studies.^19^^,^^21^^,^^24^

Interpretation should be pathway-specific and based on paired analysis. Clotting time indicates initiation, while firmness represents clot architecture.^25^^,^^26^ Fibrinolysis is assessed by comparing EX_ML with AP_ML.^27^ The difference between EX and AP lysis provides a practical measure of lysis burden and helps minimize false positives caused by preanalytical factors. Hyperfibrinolysis was rare when the EX and AP pair was used. This aligns with biological expectations in prothrombotic diseases like IMHA, PLE, and PLN and increases confidence in positive lysis calls.^1^ Dogs with IMHA have a high risk for thrombotic events.^1^ In combination with the known NETosis,^28^ it is described that similarly to our results, dogs with IMHA have reduced fibrinolysis or fibrinolysis resitance.^29^ A possible explanation is that these dogs have higher circulating levels of plasminogen activator inhibitor 1 (PAI-1) ultimately inhibiting fibrinolysis and promoting thrombosis.^30^ This hypothesis is supported by data demonstrating that these dogs have high circulating levels of PAI-1 mRNA.^31^

Disease states did not define unique viscoelastic phenotypes. Differences were present but overlap was substantial, so these metrics are most informative at the case level. In this cohort, similar to previous studies, hypercoagulability appears along a spectrum with most dogs falling near the middle and a few showing much faster and firmer clotting.^32^ The composite index combines clotting time and firmness from both main pathways, giving an overall sense of pro-coagulant activity. By adjusting for PLT and fibrin, the firmness measure highlights any extra strength not explained by these factors. If both measures are high in the same dog, it provides stronger evidence for a true procoagulant state, since 2 separate tests agree. This approach helps with risk assessment and ongoing monitoring, and it prevents overemphasis on single high results that might just be due to high platelet or fibrin levels.

Our study has several limitations. It is a retrospective, single-center study, and some disease groups had small sample sizes, which affects the strength and generalizability of our findings. In many cases, the AP channel was missing, so we could analyze lysis only in runs where both EX and AP were available, resulting in wide confidence intervals for prevalence. We did not fully standardize or record preanalytical factors, such as time from sampling to analysis, citrate fill, and temperature, which can affect lysis and firmness. The thresholds for HF and hypercoagulability were based on our cohort rather than established canine reference intervals, so they should be considered tools for phenotyping rather than diagnostic cutoffs. We did not assess clinical outcomes such as bleeding, thrombosis, or transfusion requirements, so our findings are descriptive rather than predictive. We also did not consistently include potential confounders like hematocrit, medications, and inflammatory status. The adjusted firmness model we used was simple, and using nonlinear effects or more advanced regression methods could improve our estimates. Anemia and thrombocytopenia can influence viscoelastic measurements independently of the underlying hemostatic disorder.^33^ Reduced hematocrit is associated with an apparently more hypercoagulable viscoelastic profile, whereas thrombocytopenia can reduce clot strength because platelets contribute substantially to clot formation.^3^ In our cohort, correlations between PLT and several ClotPro variables support the potential influence of hematological abnormalities on viscoelastic results. Accordingly, part of the variability observed in ClotPro variables may reflect these factors rather than disease-specific changes alone, highlighting the importance of interpreting ClotPro results in conjunction with the complete blood count.

## Off-label antimicrobial declaration

Authors declare no off-label use of antimicrobials.

## Institutional animal care and use committee or other approval declaration

Authors declare no institutional animal care and use committee or other approval was needed. The study is a retrospective design. All procedures mentioned in the study are in accordance with local legislation and institutional requirements.

## Appendix—Detailed materials and methods

This was a pilot single center retrospective study between 2022 and 2024. Dogs who had viscoelastic testing performed with the ClotPro analyzer for evaluation of coagulation disorders were included. Dogs were eligible if they had a valid ClotPro analysis including at least EX Test and IN Test assays, optimally also FIB Test and AP Test, as well as sufficient concurrent routine coagulation tests (PT, aPPT) and PLT obtained within 30 min after collection. Cases were assigned to a single primary disease category a priori based on clinician diagnosis and record review: AHDS, bleeding (non-AHDS), hyperadrenocorticism (Cushing), hepatopathy, IMHA, thrombosis of unclear etiology, PLN, PLE, or thrombocytopenia. Dogs with incomplete records, obvious sample mishandling, or duplicate measurements from the same visit were excluded. Whole blood was collected into 3.2% sodium citrate tubes of 2 mL, gently inverted, and analyzed within 30-60 min at room temperature. For each channel test, 340 μL of sample is used and the time to test completion is 60 min. ClotPro assays were run according to the manufacturer’s instructions using active-tip reagents. If present, HI-test (heparinase) results were used only for sensitivity checks and not in primary analyses. Instrument quality control follows, according to clinic standard operating procedure, biweekly and electronic checks daily. From ClotPro traces we extracted: clotting time (CT, s), amplitudes at 5/10/20 min (A5/A10/A20, mm), maximum clot firmness (MCF, mm), and maximum lysis (ML, %) for EX, IN, FIB, and AP where available. Routine laboratory variables included PT (s), aPTT (s), TT (s), PLT (×10^3^/μL), and where available D-dimer (mg/L).

### Fibrinolysis assessment

We interpreted fibrinolysis as an EX–AP pair. The primary signal was the EX_ML (%) while AP_ML (%) served as control. Then the burden metrics were calculated as ΔML = EX_ML − AP_ML and AP_MCF − EX_MCF (mm), suggesting loss of firmness due to lysis. A case was flagged as HF if (EX_ML ≥ T1 and AP_ML ≤ T2) OR (ΔML ≥ T3), with default thresholds T1 = 15%, T2 = 5%, T3 = 10% unless otherwise specified. HF prevalence analyses were restricted to runs with both EX and AP available. The rest of the cases were excluded from this analysis.

### Hypercoagulability Index and Platelet/fibrin-adjusted firmness

For the composite HCI, we combined faster initiation (shorter CT) and greater firmness (A10/MCF) across EX/IN into a single standardized score so that higher values reflect a more pro-coagulant phenotype. For each metric 𝑥 we computed a robust z-score across the cohort. We additionally estimated the portion of EX_MCF not explained by platelets and fibrin contribution (FIB_MCF) expressed as the Platelet/fibrin-adjusted firmness. Positive values indicate excess firmness beyond counts (consistent with stronger thrombin/cross-linking activity).

### Statistical analysis

Statistical analysis was performed with the R 4.5.1 and R Studio (Posit Software, PBC, Boston, MA). Normality was assessed with Q–Q plots, histograms and the Shapiro–Wilk test. Comparisons between disease groups for selected metrics (EX_CT, IN_CT, EX_MCF, IN_MCF, fibrinolysis metrics EX_ML, AP_ML, ΔML) were performed with Kruskal–Wallis test with Dunn’s post-hoc; Benjamini–Hochberg FDR adjustment was applied within each family of hypotheses. The Spearman ρ was used for correlations followed with regression analysis. HF prevalence per disease was estimated as k/n_eligible_ with exact binomial 95% CIs; denominators included only cases with both EX and AP available. Hypercoagulability was evaluated in 3 ways: (1) strict rule (high MCF and/or low CT using cohort 90th/10th percentiles), (2) HCI (continuous and ≥ 90th percentile), and (3) platelet/fibrin-adjusted residual (continuous and ≥ 90th percentile). A 2-sided FDR-adjusted *P* < .05 (*q*-value) was considered statistically significant. Given the retrospective design and small group sizes, emphasis was placed on effect sizes, CIs, and distribution overlap rather than dichotomous significance.

## Abbreviations

A10: amplitude at 10 min; A20, amplitude at 20 min
A5: amplitude at 5 min
aPTT: activated partial thromboplastin time
AP: aprotinin test (antifibrinolytic channel)
AP_MCF: maximum clot firmness in AP channel
AP_ML: maximum lysis in AP channel
BH: Benjamini–Hochberg
CI: confidence interval
CT: clotting time
DOAC: direct oral anticoagulant
ΔML: difference between EX_ML and AP_ML
EX: extrinsic pathway test
EX_A10: amplitude at 10 min in EX channel
EX_CT: clotting time in EX channel
EX_MCF: maximum clot firmness in EX channel
EX_ML: maximum lysis in EX channel
FDR: false discovery rate
FIB: fibrinogen test channel
FIB_MCF: maximum clot firmness in FIB channel
HCI: hypercoagulability index
HF: hyperfibrinolysis
HI: heparinase test
IMHA: immune-mediated hemolytic anemia
IN: intrinsic pathway test
IN_CT: clotting time in IN channel
IN_MCF: maximum clot firmness in IN channel
IQR: interquartile range
MCF: maximum clot firmness
ML: maximum lysis
NET: neutrophil extracellular traps
PAI-1: plasminogen activator inhibitor 1
PLE: protein-losing enteropathy
PLN: protein-losing nephropathy
PLT: platelet count
PT: prothrombin time
Q–Q plot: quantile–quantile plot
ROTEM: rotational thromboelastometry
s: seconds
tPA: tissue plasminogen activator
TT: thrombin time
